# Polydopamine Chelate Modified Separators for Lithium Metal Batteries with High‐Rate Capability and Ultra‐Long Cycling Life

**DOI:** 10.1002/advs.202501155

**Published:** 2025-04-01

**Authors:** Shixiang Liu, Qiang Liu, Pu Cheng, Xingkai Jia, Yinzhu Jiang, Xuan Zhang

**Affiliations:** ^1^ ZJU‑Hangzhou Global Scientific and Technological Innovation Center Zhejiang University Hangzhou 311215 P. R. China; ^2^ School of Materials Science and Engineering Zhejiang University Hangzhou 310027 P. R. China; ^3^ Institute of Nuclear Physics and Chemistry China Academy of Engineering Physics Mianyang 621900 P. R. China

**Keywords:** high‐rate capability, lithium metal batteries, polydopamine chelate, separators

## Abstract

Lithium metal batteries (LMBs) have gained significant attention because of their high theoretical energy density. However, under high‐rate charge and discharge conditions, lithium metal anodes are susceptible to dendrite formation, compromising battery safety. Creating multifunctional separators offers an effective and cost‐efficient solution for addressing fast charging and safety challenges in LMBs. This study proposes a method to prepare a functional separator by in situ growing a polydopamine copper chelate (PDA(Cu)) coating on a polypropylene (PP)/polyethylene (PE)/PP separator (PP/PE/PP@PDA(Cu)). The PDA(Cu) exhibits excellent electrolyte wetting properties and ion exclusion effects, contributing to high ionic conductivity (5.02 × 10^−^⁴ S cm^−1^) and high lithium‐ion (Li^+^) transference number (0.776). Owing to its strong adhesion to the lithium metal anode, the coating significantly suppresses the formation of lithium dendrites. The Li||Li symmetric cell with a PP/PE/PP@PDA(Cu) separator demonstrates highly stable lithium plating‐stripping cycles, lasting over 900 h. Additionally, the PDA(Cu) promotes the formation of a stable cathode electrolyte interphase (CEI) film on the LiFePO_4_ cathode surface. The LiFePO_4_||Li cell with a PP/PE/PP@PDA(Cu) separator maintains 85.1% of its capacity after 6000 cycles at 10 C. This work paves a novel path for designing separators to enhance the fast‐charging performance of LMBs and solve the challenges of lithium dendrite formation and long cycling life.

## Graphical abstract

The excellent battery performance of the  modified separator is attributed to the interactions of the polydopamine copper chelate (PDA(Cu)) coating with both the cathodes and anodes. The PDA(Cu) coating helps generate a stable cathode electrolyte interphase (CEI) film and inhibits the side reactions. The strong adhesion of the PDA(Cu) coating effectively prevents the formation of lithium dendrites.

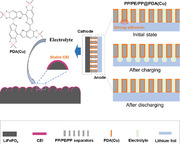

## 1 Introduction

Currently, the actual energy density of lithium‐ion batteries that use graphite anodes is close to its theoretical limit of 350 Wh kg^−^¹.^[^ ^1^ ^]^ This makes it challenging for existing lithium‐ion batteries to meet the growing demands for longer‐range electric vehicles.^[^ ^2^, ^3^ ^]^ Among the various alternative anode materials, lithium metal offers several advantages, including an extremely high specific capacity of 3860 mAh g^−1^, a very low standard redox potential of −3.040 V, and a low density.^[^ ^4^, ^5^ ^]^ Therefore, lithium metal is considered one of the most ideal anode materials. Despite the potential of lithium metal anodes, their commercialization faces significant challenges, particularly due to the formation of lithium dendrites and low Coulombic efficiency.^[^ ^6^ ^]^ The interface between lithium metal and the electrolyte constantly undergoes reactions, leading to a low Coulombic efficiency. This issue is exacerbated at high current densities, primarily due to the uneven deposition of lithium metal, leading to the formation of lithium dendrites.^[^ ^7^ ^]^ To enhance the cycling performance and safety of lithium metal batteries, it is essential to protect the surface of the lithium metal anode, slow down the reaction between the lithium metal and the electrolyte, and minimize the formation of lithium dendrites.

To address the aforementioned issues, researchers have employed strategies such as optimizing electrolyte compositions,^[^ ^8^, ^9^, ^10^ ^]^ developing solid‐state electrolytes,^[^ ^11^, ^12^ ^]^ constructing artificial solid electrolyte interphase (SEI) films,^[^ ^13^, ^14^, ^15^ ^]^ and designing 3D lithium deposition frameworks^[^ ^16^, ^17^, ^18^ ^]^ to regulate lithium deposition behavior. However, these methods are challenging to implement in the mass production of lithium metal batteries due to the failure tendency of additives, the high costs of solid‐state electrolytes, the high reactivity of lithium, and strict operating conditions. Therefore, simpler and cheaper strategies need to be developed. The surface modification of separators is an effective way to prevent the formation of lithium dendrites.^[^ ^19^, ^20^, ^21^ ^]^ The structure and performance of separators are crucial for stable lithium plating and stripping. Developing high‐performance separators with special designs can guide the uniform deposition of lithium ions. In addition, surface modification can improve the wettability between the separators and the electrolyte, as well as enhance the thermal stability of the separators. Surface modification of the separators can be divided into inorganic coating modification and organic coating modification. Inorganic coating modification involves applying inorganic nanoparticles such as SiO_2_,^[^ ^22^ ^]^ Al_2_O_3_,^[^ ^23^ ^]^ and boehmite^[^ ^24^ ^]^ to both sides of the polyolefin separator using a binder. However, the coatings on separators modified with inorganic materials have poor adhesion,^[^ ^25^ ^]^ and they may detach from the separator substrate during battery assembly and charge–discharge cycles.^[^ ^26^ ^]^ This detachment can significantly compromise the structural integrity of the separator and lead to a rapid decline in the overall performance and lifespan of the battery. Furthermore, the relatively high density of inorganic materials affects the lightweight nature of the separator. Compared to inorganic coatings, polymer coatings have a strong adhesive structure^[^ ^27^ ^]^ that can form a flexible and continuous layer, ensuring a more durable contact with the electrode. Additionally, polymers have a lower density. Therefore, organic polymer coatings have long been favored by researchers in the field of lithium metal batteries.

Polydopamine (PDA) is a novel polymer synthesized inspired by the adhesive proteins found in mussels and is widely used for the surface modification of various materials.^[^ ^28^ ^]^ PDA contains polar functional groups such as amine and catechol. These compositions provide it with strong adhesion to various substrates and a good affinity for polar solvents.^[^ ^29^ ^]^ Dopamine can undergo oxidative self‐polymerization to form a submicron‐thick coating on the separator surface. The ultrathin PDA coatings have minimal impact on the pore structure of the separator. PDA coatings can enhance the wettability between separators and polar electrolytes^[^ ^30^ ^]^ while also adhering the separator to the lithium metal anode to suppress the formation of lithium dendrites.^[^ ^31^, ^32^, ^33^ ^]^ However, PDA is a polymer formed by the combination of covalent and non‐covalent bonds.^[^ ^34^, ^35^ ^]^ It tends to depolymerize into small molecules in polar electrolytes,^[^ ^36^, ^37^ ^]^ which may reduce the ionic conductivity of the electrolyte. When applying PDA in batteries, it is essential to address the problem of its chemical structural instability in polar electrolytes.

In this study, we took advantage of the adhesive properties of PDA and its ability to chelate metal ions^[^ ^38^ ^]^ to in situ grow a PDA copper chelate (PDA(Cu)) coating on a polypropylene (PP)/polyethylene (PE)/PP separator (PP/PE/PP@PDA(Cu)). This process effectively replaced the originally unstable hydrogen bonds and *π*–*π* stacking interactions of PDA with chemically stable coordination bonds. The resulting PP/PE/PP@PDA(Cu) separators exhibit excellent electrolyte resistance, electrolyte wettability, and thermal stability. Additionally, the PDA(Cu) coating can greatly enhance the ion conductivity, Li^+^ transference number, and electrochemical stability of PP/PE/PP separators. It also effectively inhibits the formation of lithium dendrites and encourages the development of stable CEI films on the surface of the cathode. Compared to batteries using polyolefin separators, LiFePO_4_||Li batteries with PP/PE/PP@PDA(Cu) separators exhibit significantly improved cycling and rate performance.

## 2 Results and Discussion

### 2.1 Morphology and Structure of Separators

**Figure 1:**
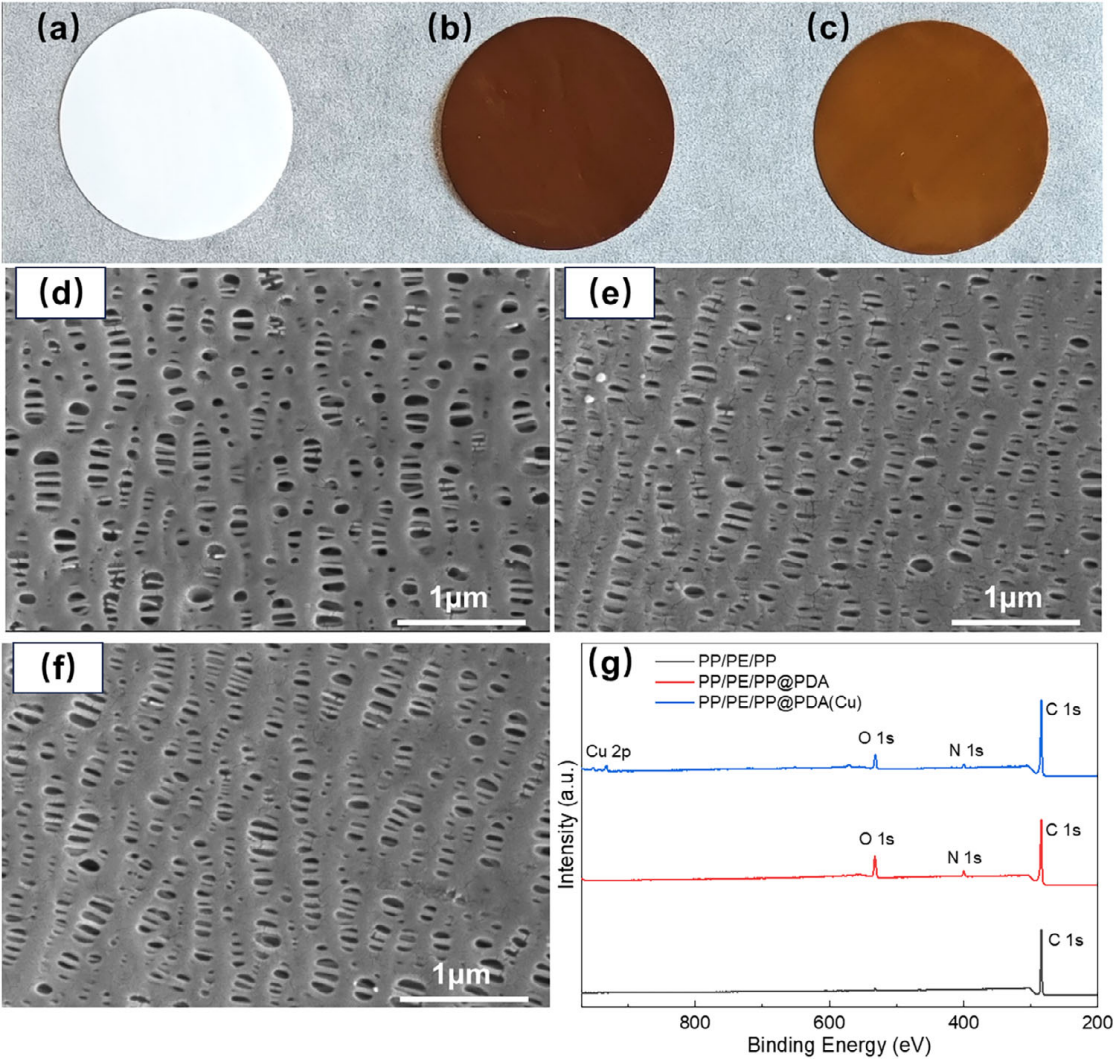
Digital photos of a) PP/PE/PP; b) PP/PE/PP@PDA; c) PP/PE/PP@PDA(Cu). SEM photographs of d) PP/PE/PP; e) PP/PE/PP@PDA; f) PP/PE/PP@PDA(Cu). g) Full XPS spectra of different separators.

In this study, PP/PE/PP separators were modified by the self‐assembly of PDA and PDA chelate, respectively. After the modification, the color of PP/PE/PP separators changed from white to dark brown (PP/PE/PP@PDA) and brown (PP/PE/PP@PDA(Cu)), respectively (**Figure** **1**a–c). The XPS results (Figure 1g) can further confirm PDA and PDA(Cu) coating onto PP/PE/PP separators. Compared with PP/PE/PP separators, there are two new peaks for PDA‐treated separators that emerged at 399.6 eV (N 1s) and 532.4 eV (O 1s), which come from the catechol group and amino group of PDA coating. In contrast to PDA‐treated separators, the Cu 2p peaks (932.5 and 952.4 eV) appear on the full XPS spectrum of PDA(Cu)‐treated separators, and the peak of O 1s changes from 532.4 to 531.5 eV (Figure S1, Supporting Information). The decrease in binding energy of O 1s is due to the formation of *π*‐back bonding of Cu(II) with C═O, which increases the electron cloud density of the coordinating atoms in the ligand.^[^ ^39^, ^40^ ^]^ Figure S2 (Supporting Information) shows the EPR results of PP/PE/PP@PDA and PP/PE/PP@PDA(Cu). PDA exhibits stable semiquinone radicals, resulting in a strong peak in the EPR spectrum of PP/PE/PP@PDA at 3510 G. However, there is no obvious semiquinone radical signal that can be seen in the EPR spectrum of PP/PE/PP@PDA(Cu). It's due to the chelation reaction between semiquinone radicals and Cu^2+^ (Figure S3, Supporting Information). As shown in Figure 1d–f, the pore structures of the separators change little even after the modification by PDA or PDA(Cu). To further reveal the effect of coating modification on the pore structure of separators, the air permeances of separators were tested. Figure S4 (Supporting Information) displays the air permeances of separators by the Gurley method. Compared with PP/PE/PP, the Gurley number of PP/PE/PP@PDA(Cu) only increased by 8.0%, but that of PP/PE/PP@PDA increased by 41.1%. The deterioration of air permeances of PP/PE/PP@PDA is likely due to its thicker coating thickness (Figure S5, Supporting Information).

### 2.2 Physical and Chemical Properties of Separators

**Figure 2:**
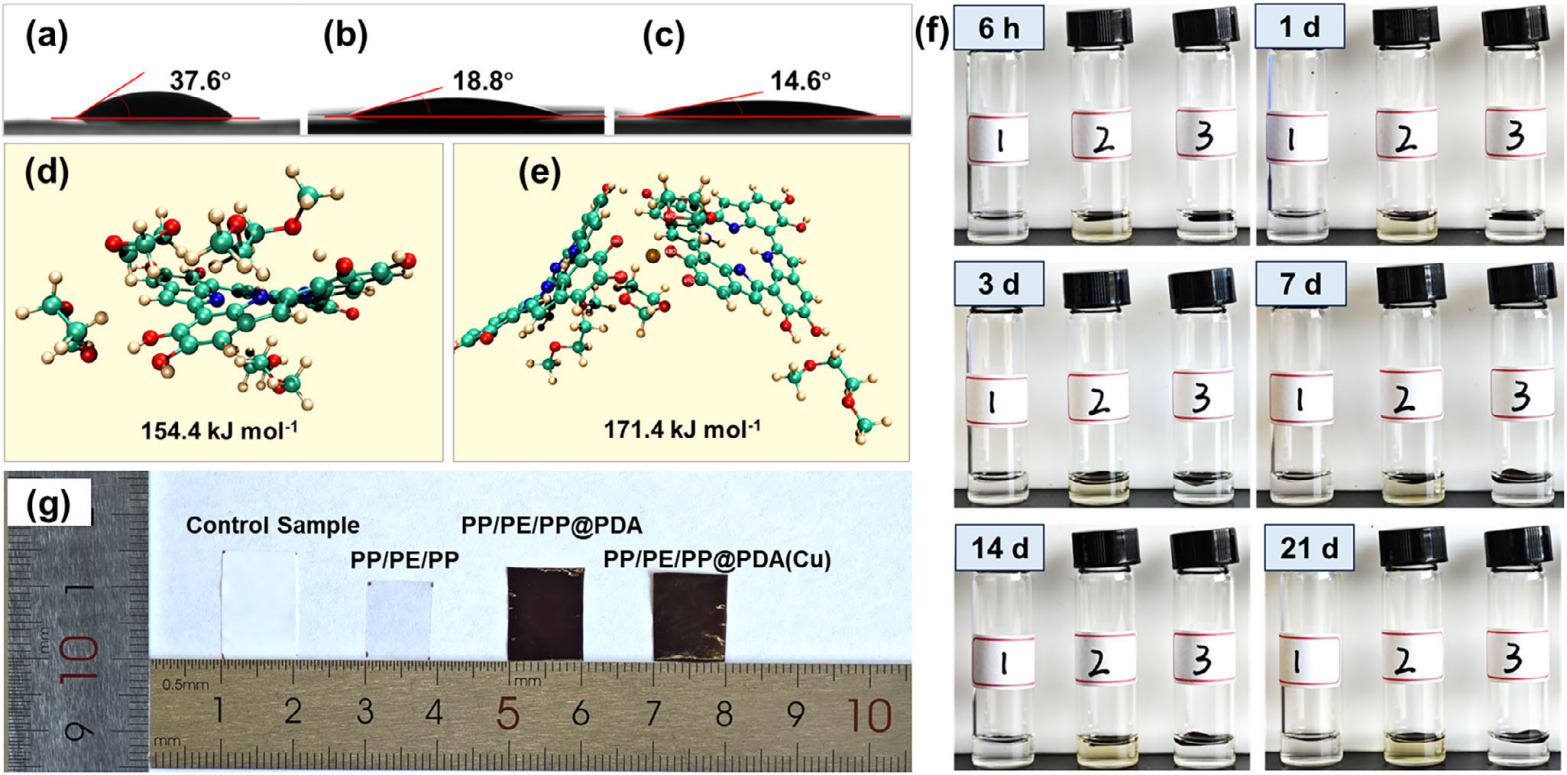
Contact angle images of a) PP/PE/PP; b) PP/PE/PP@PDA; c) PP/PE/PP@PDA(Cu). The adsorption energy through DFT calculations between DOL‐DME and d) PDA; e) PDA(Cu). f) Digital photographs of (1) PP/PE/PP, (2) PP/PE/PP@PDA, and PP/PE/PP@PDA(Cu) after the immersion in 1 mol·L^−1^ LiTFSI in the electrolyte (DOL: DME = 1:1, 2wt.% LiNO_3_) at different time points. g) Shrinkage test of PP/PE/PP, PP/PE/PP@PDA, and PP/PE/PP@PDA(Cu) at 160 °C for 30 min.

**Figure 3:**
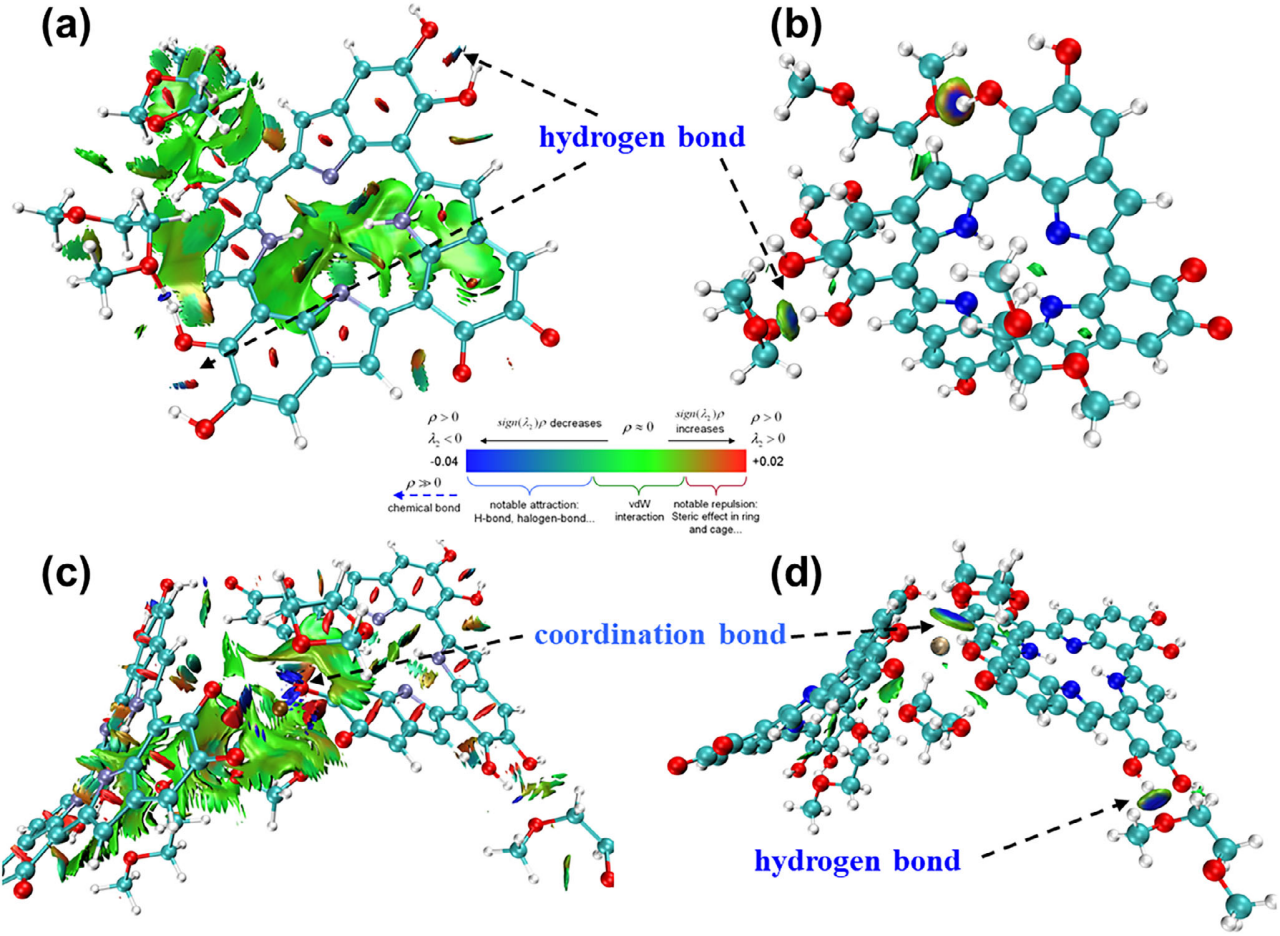
Weak interaction analysis: interaction region indicator (IRI) of the model structure of a) PDA and c) PDA(Cu) without covalent bond interaction, intrafragment interactions revealed by independent gradient model (IGM) based on Hirshfeld partition of the model structure of b) PDA and d) PDA(Cu).

The wettability of the separator plays a crucial role in the performance of lithium‐ion batteries. For efficient Li^+^ transfer, the separator positioned between the anode and cathode must ensure complete contact with the electrolyte while also maintaining a long‐lasting ability to retain the electrolyte.^[^ ^41^ ^]^ Otherwise, it will increase the internal resistance and reduce the performance of batteries. The value of the contact angle (CA) between the separator surface and electrolytes can directly reflect the wettability. As shown in **Figure** **2**a–c, the CA of PP/PE/PP, PP/PE/PP@PDA, and PP/PE/PP@PDA(Cu) are 37.6°, 18.8°, and 14.6°, respectively. Due to the absence of polar groups in the polyolefin molecular chain structure, the PP/PE/PP has a poor affinity with polar electrolytes,^[^ ^42^ ^]^ which results in a larger CA. The CA of PP/PE/PP@PDA decreases significantly because of the polar groups of PDA. In contrast to PP/PE/PP@PDA, the CA of PP/PE/PP@PDA(Cu) declines further. It is ascribed to the stronger interaction between PDA(Cu) and electrolytes, which can be proved by DFT calculations (Figure 2d,e). The electrolyte is composed of 1,3‐dioxolane (DOL) and 1,2‐dimethoxyethane (DME). The adsorption energy value between DOL/DME and PDA(Cu) (171.4 kJ mol^−1^) is larger than that between DOL/DME and PDA (154.4 kJ mol^−1^). To understand the origin of the enhanced weak interactions between the electrolyte and PDA(Cu), the interaction region indicator (IRI) and intrafragment interactions were calculated using the independent gradient model (IGM) through Multiwfn with the wavefunction files. **Figure** **3** shows that both leading van der Waals interaction and intermolecular hydrogen bond interaction contribute to the wettability of PDA and PDA(Cu) model systems. The intramolecular hydrogen bond interaction in the PDA organic moiety was also observed in the PDA and PDA(Cu) model systems. Moreover, the Cu^2+^ in PDA(Cu) can form a coordination bond with DOL solvent (Figure 3c,d), which can be further confirmed by the electron localization function (ELF) (Figure S6, Supporting Information), Mayer bond order, and fuzzy bond order (Table S1, Supporting Information) of the Cu‐center structure in PDA(Cu). By localized molecular orbital (LMO) (Figure S7, Supporting Information) and orbital composition analysis, it manifests that the coordination bonds between the PDA skeleton and Cu^2+^ predominantly consist of the hybridized orbital of Cu^2+^ (4s, 4p, and 4d) and conjugated *π* orbital delocalized on the PDA skeleton. Hence, the excellent electrolyte wettability of PP/PE/PP@PDA(Cu) benefits from the strong hydrogen bond interaction and coordination bond interaction between PDA(Cu) and the electrolyte.

The chemical stability of coating on PP/PE/PP separators in electrolytes can affect the performance of lithium batteries. Figure 2f displays the color change of electrolytes after soaking the unmodified and modified separators with time. The color of the electrolyte with PP/PE/PP@PDA changes from clear to brown and deepens over time. This is probably because PDA contains a large number of non‐covalent structures that are unstable in polar organic solvents. The depolymerization of PDA results in numerous small molecular fragments that dissolve in the electrolyte. However, the color of the electrolyte with PP/PE/PP@PDA(Cu) has always been clear, even after 21 days. It is due to the oligomer fragment of PDA chelated with copper ion instead of the *π*–*π* stacking interactions, which endows PDA(Cu) with excellent chemical stability in the electrolyte.

Polyolefin separators are prone to shrinkage at high temperatures, which will cause direct contact between the anode and cathode. This contact may result in a short circuit, compromising the safety of the battery. As shown in Figure 2g, the thermal stability between unmodified and modified separators is compared. The dimensional shrinkage of the unmodified PP/PE/PP separator is 41.3% after thermal treatment at 160 °C for 30 min. By contrast, the dimensional shrinkage of PP/PE/PP@PDA(Cu) is only 19.3%. The dimensional shrinkage of PP/PE/PP@PDA (13.3%) is slightly better than that of PP/PE/PP@PDA(Cu), which is due to a thicker coating. In general, PDA and PDA(Cu) can form thermostable support structures on the surface and within the pores of PP/PE/PP separators to greatly improve their thermal stability.

### 2.3 Electrochemical Properties of Separators

**Figure 4:**
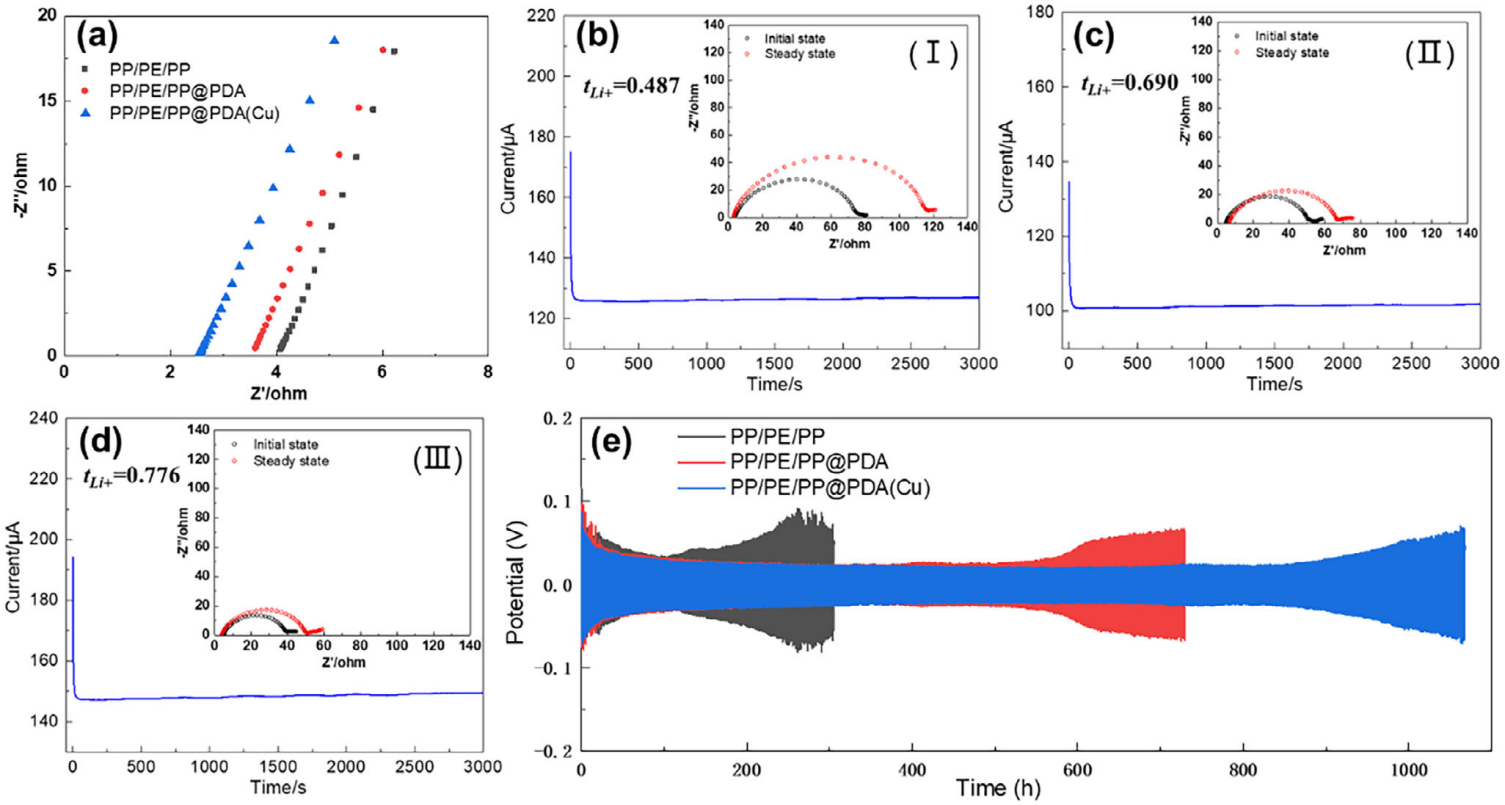
a) Nyquist plots of symmetrical SS||SS cells. Chronoamperometry of Li||Li cells at 25 °C assembled with b) PP/PE/PP; c) PP/PE/PP@PDA; d) PP/PE/PP@PDA(Cu). Inset: EIS for the same cells before and after polarization assembled with (I) PP/PE/PP; (II) PP/PE/PP@PDA; (III) PP/PE/PP@PDA(Cu). e) Li||Li cells with various separators at 0.5 mAh cm^−2^ and at 0.5 mA cm^−2^.

Ionic conductivity is an important parameter of the separator, which reflects the ease of transporting anions and cations in the separator. **Figure** **4**a displays the ionic resistance of stainless steel (SS) symmetric cells assembled with the pristine and modified PP/PE/PP separators. The ionic conductivities of PP/PE/PP, PP/PE/PP@PDA, and PP/PE/PP@PDA(Cu) calculated from the ionic resistance are 0.312, 0.353, and 0.502 mS cm^−1^, respectively. The ionic conductivity of separators is primarily influenced by their electrolyte wettability and pore structures. Due to the excellent electrolyte wettability and similar air permeances with pristine PP/PE/PP separators, PP/PE/PP@PDA(Cu) has the best ionic conductivity of the three kinds of separators. Although PP/PE/PP@PDA also has excellent electrolyte wettability, its ionic conductivity is only slightly improved compared to PP/PE/PP. In addition to reduced air permeability, the poor chemical stability of coatings in electrolytes may also be the reason that hinders the increase of ionic conductivity of PP/PE/PP@PDA. To investigate the effect of small molecules produced by the decomposition of PDA in the electrolyte on ionic conductivity, the ionic resistance of the SS||SS cell assembled with PP/PE/PP separators and electrolytes with small molecules of PDA was tested (Figure S8, Supporting Information). Compared with the PP/PE/PP separators with pristine electrolytes, the ionic conductivity of the PP/PE/PP separators with electrolytes containing the small molecules decreases to 0.177 mS cm^−1^. This is likely because the small molecule impurities dissolved in the electrolyte increase the concentration of the electrolyte, which hinders the diffusion of ions.

The ionic conductivity of separators is influenced by both cations and anions. However, only the movement of Li^+^ is significant for battery performance. Therefore, it is essential to investigate the Li^+^ transference number of different separators. The Li^+^ transference number calculated by the combination of chronoamperometry and EIS of Li||Li symmetric cells is displayed in Figure 4b–d. The lithium‐ion transference numbers of PP/PE/PP, PP/PE/PP@PDA, and PP/PE/PP@PDA(Cu) are 0.487, 0.690, and 0.776, respectively. Compared with the PP/PE/PP, the modified separators have a higher lithium ions transference number, which is owing to the ─NH─ groups of PDA and PDA(Cu) having strong interaction with TFSI^−^ to release more free lithium ions.^[^ ^30^ ^]^ Due to the poor chemical stability of PDA in the electrolytes, small molecules dissolved in the electrolyte adsorb TFSI^−^ and continue to migrate in the electrolyte under the action of an electric field, resulting in the Li^+^ transference number of PP/PE/PP@PDA lower than that of PP/PE/PP@PDA(Cu).

The electrochemical stability of separators can be assessed using the linear sweep voltammetry (LSV) method. As can be seen in Figure S9 (Supporting Information), the electrochemical window of PP/PE/PP separators is 4 .60V. By comparison, the decomposition potential of PP/PE/PP@PDA and PP/PE/PP@PDA(Cu) separators is up to 4.62 and 4.63 V, respectively. The improved electrochemical stability of modified separators results from enhanced wettability, allowing for greater electrolyte retention in the separators and minimizing the contact between free solvent molecules and the cathode to prevent the oxidative decomposition of the electrolyte.

The cycling performance of Li||Li symmetric cells was utilized to assess the effectiveness of separators in preventing the dendritic growth of lithium. Figure 4e presents the voltage versus time curves of symmetric cells at 0.5 mAh cm^−2^ and at 0.5 mA cm^−2^. In the initial stage of the cycle, the continuous reduction in overpotential for Li||Li symmetric cells can be attributed to the activation of the electrodes. However, as the cycling time increases, the overpotential gradually rises. This rise is due to the formation of a dead lithium layer, which thickens over time, creating a tortuous diffusion path for lithium ions and leading to an increase in cell resistance. The cell with a PP/PE/PP separator exhibits an obvious increase in polarization voltage after 120 h of cycling. In comparison, the stable cycling time of cells with PP/PE/PP@PDA and PP/PE/PP@PDA(Cu) is 590 and 900 h, respectively. The impedances of the Li||Li cells before and after cycling with different separators (Figure S10, Supporting Information) were tested to further investigate the role of PP/PE/PP@PDA(Cu) separators in enhancing interfacial stability. For the Li||Li symmetric cell assembled with PP/PE/PP, the *R*_ct_ first decreases and then increases during 140 cycles. The decrease in *R*_ct_ may be due to the activation of the electrode, which formed an SEI layer with high ionic conductivity. The increase in *R*_ct_ was likely caused by side reactions at the interface that disrupted the SEI layer. For the Li||Li symmetric cell assembled with PP/PE/PP@PDA(Cu), the *R*_ct_ continuously decreased and eventually stabilized. Moreover, the *R*_ct_ before and after cycling for the PP/PE/PP@PDA(Cu)‐assembled Li||Li symmetric cell is lower than that for the PP/PE/PP‐assembled Li||Li symmetric cell. These results suggest that PP/PE/PP@PDA(Cu) can suppress side reactions and the formation of lithium dendrites during the cycling process.

### 2.4 Battery Performance

**Figure 5:**
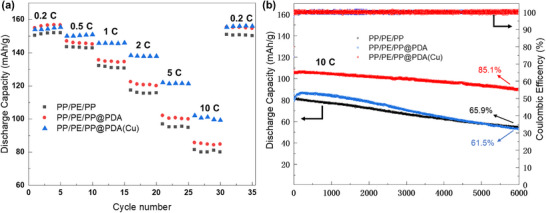
a) Rate performance of LiFePO_4_||Li cells with various separators. b) Long‐term charge and discharge cycling of LiFePO_4_||Li cells with various separators at 10 C.

In view of the excellent physicochemical and electrochemical properties of PP/PE/PP@PDA(Cu), the rate and cycle performance of the LiFePO_4_||Li cells assembled with it were further investigated. As shown in **Figure** **5**a, the battery with PP/PE/PP@PDA(Cu) behaves better rate performance, especially at high C‐rate, the discharge capacities are 155, 151, 146, 138, 122, and 101 mAh g^−1^ at 0.2, 0.5, 1, 2, 5 and 10 C, respectively. In comparison to the battery with PP/PE/PP, the discharge capacity of the battery with PP/PE/PP@PDA(Cu) at 10 C increases by 26.3%, which benefits from the higher ionic conductivity and Li^+^ transference number of PP/PE/PP@PDA(Cu). Furthermore, the long‐term cycling stability of the cells with pristine and modified separators at 10 C was tested. As shown in Figure 5b, although the discharge capacity of the battery with PP/PE/PP@PDA is higher than that of the battery with PP/PE/PP in the early and middle stages of cycling, its capacity fading rate is faster. This may be due to the small molecules produced by PDA decomposition, which significantly increase the viscosity of the electrolyte as it is consumed during cycling. This leads to a rapid decline in the battery's electrochemical performance. Among these three types of batteries, the battery with PP/PE/PP@PDA(Cu) demonstrates the highest capacity and the best cycling stability, and its capacity retention rate remains as high as 85.1% even after 6000 cycles. Compared to the LiFePO_4_||Li cells developed in recent years, the cells assembled in this study have a significant advantage in capacity retention during high‐rate cycling (**Table** **1**).

**Table 1:** Summary of electrochemical performances of LiFePO_4_||Li batteries with various separators.

Separator material	C‐rate	Capacity retention	Refs.
PVDF‐HFP/UiO‐66‐NH_2_	1 C	86.7% /100 cycles	[43]
PP/SiO_2_ aerogel	1 C	87.4% /100 cycles	[22]
Fluorinated PEEK	0.5 C	93% /300 cycles	[44]
PP/ZIF8/ILs	0.2 C	82.6% /450 cycles	[45]
Phenolic resin/PE	0.4 C	86% /450 cycles	[46]
PVDF‐HFP/UN‐SLi/GF	5 C	80% /3000 cycles	[47]
PP/PE/PP@PDA(Cu)	10 C	85.1% /6000 cycles	This work

**Figure 6:**
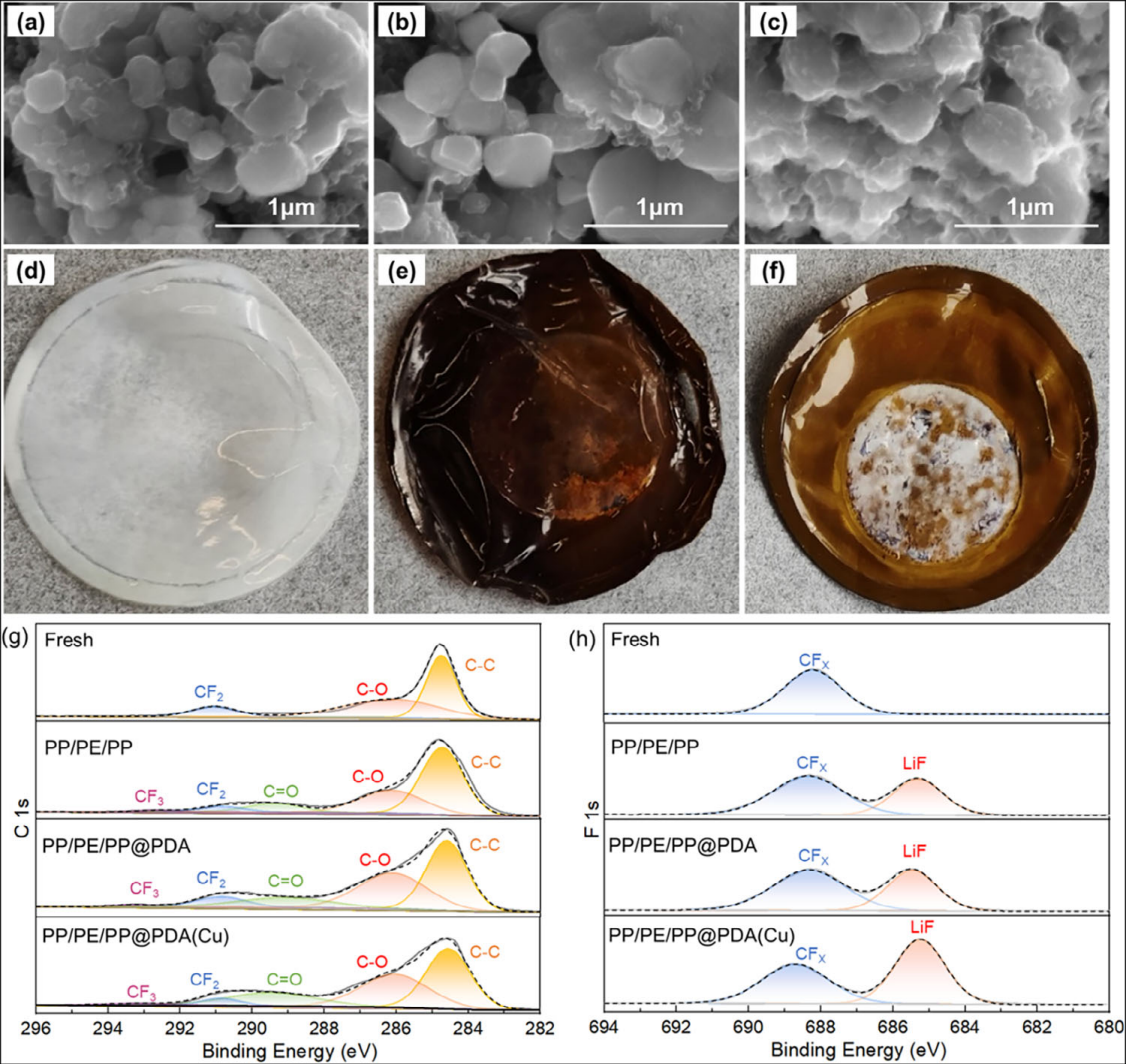
SEM photographs of LiFePO_4_ cathode with various separators (a) PP/PE/PP; b) PP/PE/PP@PDA; c) PP/PE/PP@PDA(Cu)) after 6000 cycles. Digital photographs of various separators on the cathode side after 6000 cycles: d) PP/PE/PP; e) PP/PE/PP@PDA; f) PP/PE/PP@PDA(Cu). XPS spectra of the LiFePO_4_ harvested from LiFePO_4_||Li cells with various separators after 6000 cycles: g) C 1s spectra; h) F 1s spectra.

To further reveal the potential mechanism of the excellent cycling stability of the battery with PP/PE/PP@PDA(Cu) at high C‐rate, the battery components obtained by disassembling the recycled batteries have been studied. **Figure** **6** displays the morphology of cathode materials and separators on the cathode side after 6000 cycles. In contrast to the LiFePO_4_ particles of batteries with PP/PE/PP or PP/PE/PP@PDA, the LiFePO_4_ particles of batteries with PP/PE/PP@PDA(Cu) are rougher (Figure 6a–c). Meanwhile, the area where the PP/PE/PP@PDA(Cu) contacts the cathode turned white (Figure 6f). This is likely because the PDA(Cu) coating reacted with the cathode to form a stable CEI film.^[^ ^48^ ^]^ The EDS results of the LiFePO_4_ cathode with PP/PE/PP@PDA(Cu) (Figures S11 and S12, Supporting Information) indicate that the CEI contains Cu and N, which is consistent with the chemical composition of PDA(Cu). XPS analysis (Figure 6g,h) was carried out to obtain the molecular structures of the CEI film on the post‐cycled cathodes. After 6000 cycles at 10 C, three new peaks appear on the surface of LiFePO_4_ cathodes, corresponding to CF_3_ (293.1 eV), C═O(289.2 eV), and LiF (685.3 eV), respectively. It is worth noting that a much stronger LiF signal appears on the LiFePO_4_ cathode of batteries with PP/PE/PP@PDA(Cu). It means the PDA(Cu) coating helps generate a stable CEI film rich in LiF and inhibits the side reactions between the cathode and electrolyte (Figure 8), which helps improve the cycle life of batteries.

**Figure 7:**
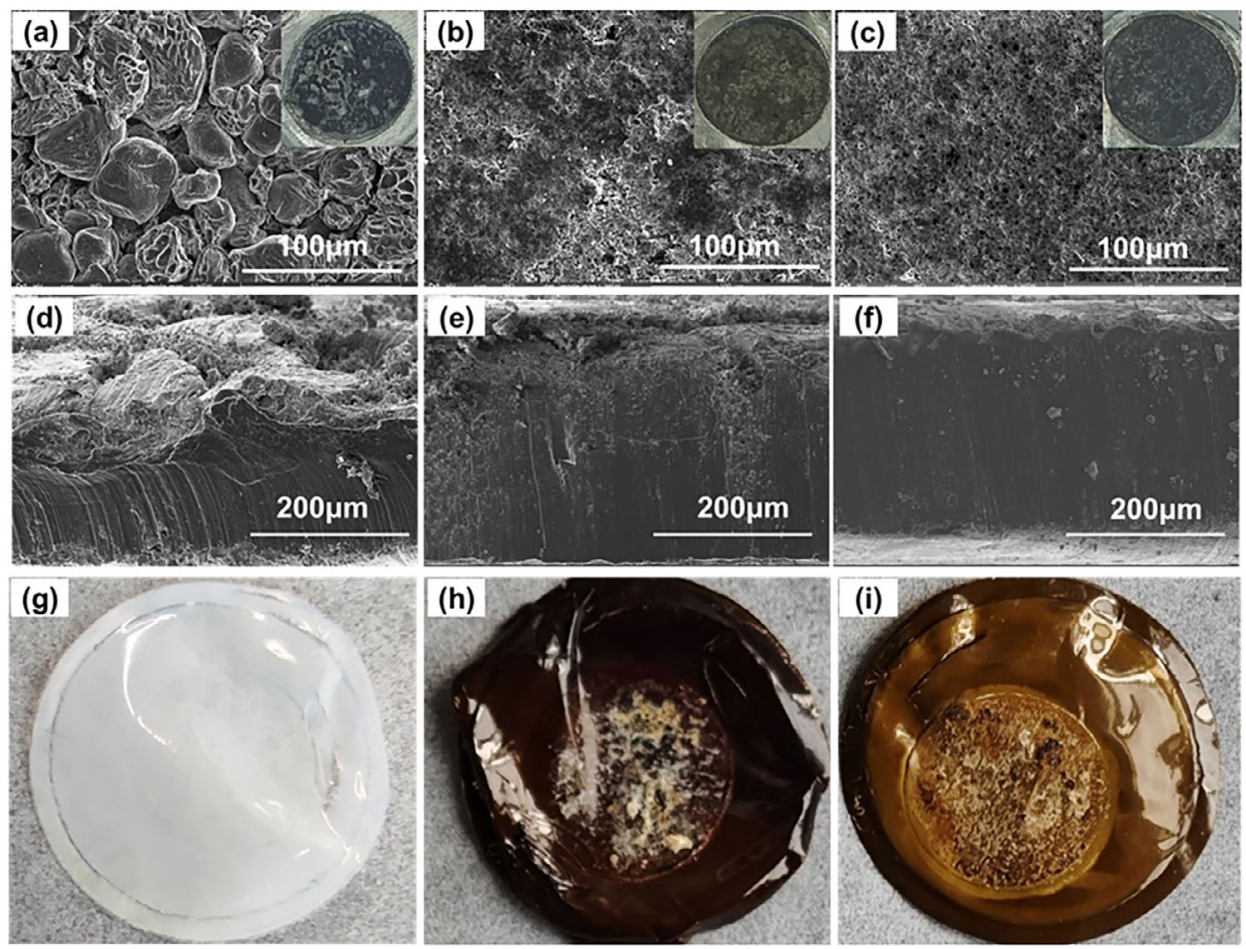
The surface SEM photographs and digital photographs (inset) of Li anode with various separators (a) PP/PE/PP; b) PP/PE/PP@PDA; c) PP/PE/PP@PDA(Cu)) after 6000 cycles. The cross‐sectional SEM photographs of Li anode with various separators (d) PP/PE/PP; e) PP/PE/PP@PDA; f) PP/PE/PP@PDA(Cu)) after 6000 cycles. Digital photographs of various separators on the anode side after 6000 cycles: g) PP/PE/PP; h) PP/PE/PP@PDA; i) PP/PE/PP@PDA(Cu).

Additionally, the uncontrolled deposition of lithium dendrites on the anode can result in a shorter cycle life and pose serious safety risks in LMBs. **Figure** **7** shows the morphology of the Li anode and separators on the anode side after 6000 cycles. Many block‐shaped lithium dendrites appear on the anode surface of the battery with PP/PE/PP (Figure 7a). However, almost no lithium dendrites were found on the anode surface of batteries with PP/PE/PP@PDA or PP/PE/PP@PDA(Cu) (Figure 7b,c). The cross‐sectional SEM photographs further reveal that the lithium anode of the battery with PP/PE/PP has formed a large number of lithium dendrites and dead lithium (Figure 7d), while the cross‐section of the lithium anode of the battery with PP/PE/PP@PDA(Cu) remains dense and flat (Figure 7f). The separators on the anode side were investigated to further reveal this difference in the anode surface (Figure 7g–i). The surface of PP/PE/PP is clear, while the surface of PP/PE/PP@PDA and PP/PE/PP@PDA(Cu) has a lot of lithium debris. The strong adhesion of the modified coating to the lithium foil is likely the key factor that leads to uniform lithium deposition.

**Figure 8:**
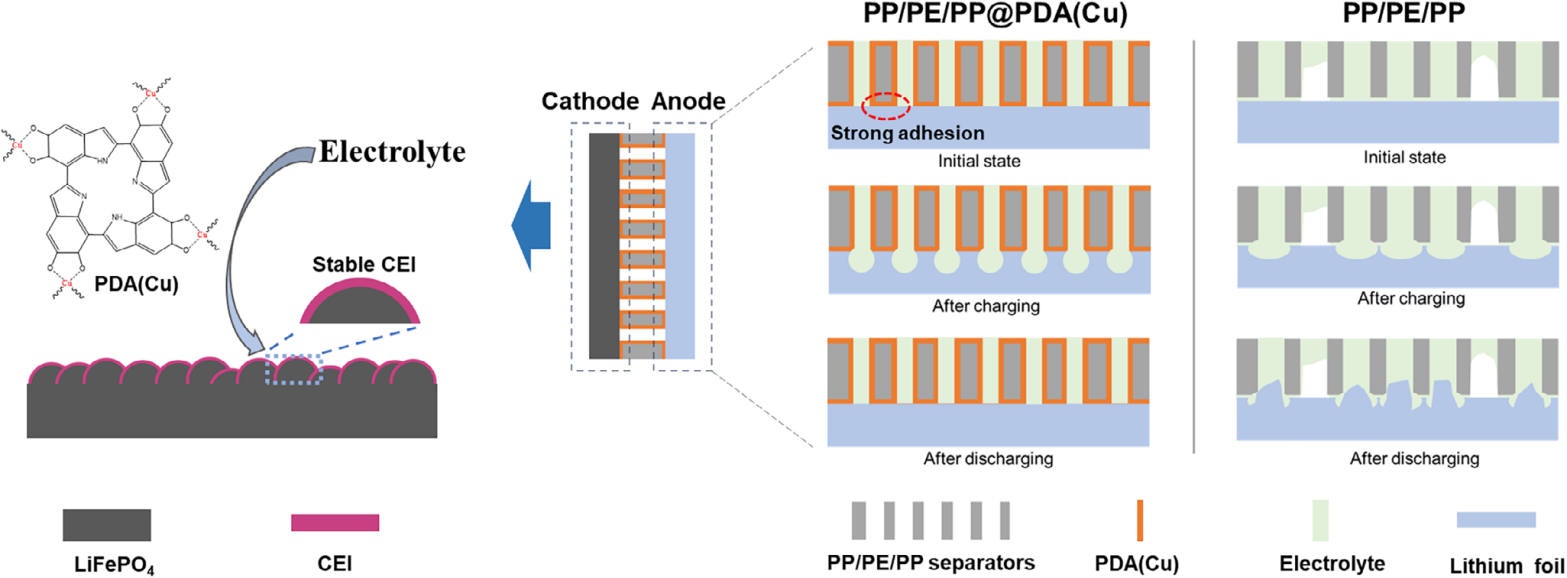
Schematic illustration for the working principle of PP/PE/PP@PDA(Cu) separator in promoting the formation of stable CEI and suppressing lithium dendrites.

Due to the loose bonding between the PP/PE/PP and the lithium foil, there is a freely flowing electrolyte between the non‐porous sections of the separator and the lithium foil. During the charging process, lithium stripping occurs at both the lithium foil corresponding to the porous sections of the separator and the lithium foil corresponding to the non‐porous sections. However, during the discharging process, lithium preferentially deposits in the areas corresponding to the porous sections of the separator, making it difficult for lithium to deposit in the areas corresponding to the non‐porous sections. This uneven deposition leads to the formation of a significant amount of lithium dendrites.^[^ ^49^, ^50^ ^]^ For PP/PE/PP@PDA(Cu), the strong adhesion of catecholic in its coating makes it difficult for the adhered lithium foil surface to contact the electrolytes (**Figure** **8**). During the charging/discharging process, lithium stripping/deposition primarily occurs only in the areas of the lithium foil corresponding to the porous sections of the separator. The process of lithium stripping and deposition is highly reversible, which makes it challenging for lithium dendrites to develop.

## 3 Conclusion

In summary, the PDA(Cu) coating endows PP/PE/PP separators with superior electrolyte wettability, thermal stability, and electrochemical stability. The PP/PE/PP@PDA(Cu) has an ion transference number of 0.776 and an ionic conductivity of 5.02 × 10^−4^ S cm^−1^ at room temperature. The Li||Li symmetrical batteries assembled using this separator can stably cycle for 900 h at a current density of 0.5 mA cm^−2^. Furthermore, the LiFePO_4_||Li batteries with this separator maintain a capacity retention of 85.1% after 6000 cycles at a rate of 10 C. The excellent battery performance is attributed to the exceptional adhesion of PDA(Cu) to the anode and the promotion function of PDA(Cu) in the generation of a stable CEI film. This work addresses the issue of polydopamine's instability in electrolytes within battery applications while also opening a new pathway for the ultra‐long cycling use of lithium metal batteries at high rates.

## 4 Experimental Section

Information regarding sample preparation, characterization, and electrochemical measurements is available in the Supporting Information.

## Conflict of Interest

The authors declare no conflict of interest.

## Supporting information



Supporting Information

## Data Availability

The data that support the findings of this study are available from the corresponding author upon reasonable request.
