# Supplementary material for: Polydopamine Chelate Modified Separators for Lithium Metal Batteries with High‐Rate Capability and Ultra‐Long Cycling Life
Source: Adv Sci (Weinh). 2025 Apr 1;12(25):2501155. doi: 10.1002/advs.202501155 (PMC12224971; doi:10.1002/advs.202501155)
Supplement: Supplementary file 1 — Supporting Information [file ADVS-12-2501155-s001.docx]

**Supporting Information**

Polydopamine Chelate Modified Separators for Lithium Metal Batteries with High-Rate Capability and Ultra-long Cycling Life

Shixiang Liu^a,b^, Qiang Liu^c^, Pu Cheng^a,b^, Xingkai Jia^b^, Yinzhu Jiang^a,b,*^, Xuan Zhang^a,b,*^

a ZJU‑Hangzhou Global Scientific and Technological Innovation Center, Zhejiang University, Hangzhou 311215, PR China

b School of Materials Science and Engineering, Zhejiang University, Hangzhou 310027, PR China

c Institute of Nuclear Physics and Chemistry, China Academy of Engineering Physics, Mianyang 621900, PR China

**Experimental Section**

*Synthesis of* *PDA(Cu) Functionalized Separators*: 6.1 g of Trizma base (Aladdin), 5.3 g of copper(II) sulfate pentahydrate (CuSO_4_•5H_2_O, Aladdin), and 4.0 g of ammonium persulfate ((NH_4_)_2_S_2_O_8_, Aladdin) were added to 1000 ml of deionized water. After the reagents were completely dissolved with stirring, the pH of the solution was adjusted to 8.5 using dilute hydrochloric acid, and then 2.0 g of dopamine hydrochloride (DA, Aladdin) was added. The solution was stirred for 10 minutes to obtain the precursor for modifying the separators. The PP/PE/PP separator (Celgard 2325) was added to the precursor solution and let stand at 25°C for 12 hours, resulting in the in situ modified polyolefin separator with polydopamine copper chelate (PP/PE/PP@PDA(Cu)). The PP/PE/PP@PDA(Cu) was washed with deionized water four times and then placed in a vacuum oven at 50°C to be dried for 24 hours. The preparation method of the polyolefin separator with polydopamine (PP/PE/PP@PDA) was the same as above, except that CuSO_4_•5H_2_O was not added.

*Preparation of the electrolyte with PDA depolymerization products (PDADP)*: The PP/PE/PP@PDA separators were put into 1 ml of electrolyte (1 M LiTFSI in DOL/DME with 2 wt.% LiNO_3_) and let stand for one week. Then, the PP/PE/PP@PDA separators were removed, and the residual electrolyte was used to investigate how PDADP affects the ionic conductivity of the separators.

*Characterization*: The morphologies of the separators, LiFePO_4_ particles, and lithium foil were characterized by using SEM (Thermo Fisher Scientific, Scios2 Hivac). The X-ray photoelectron spectroscopy (XPS) experiments were carried out by a K-Alpha XPS spectrometer (Thermo Scientific). Electron paramagnetic resonance (EPR) spectra of modified separators at ambient temperature were collected on an EMXplus spectrometer (Bruker) at a microwave power of 0.2 mW. The contact angles of the separators were obtained by the contact angle meter (Lauda Scientific LSA100). The air permeances of the separators were tested by an air permeability tester (Paratronix, PAPT-B01).

*Ion Transport Properties Tests*: The electrochemical performance of the separators was characterized using coin cells (CR2025) assembled in an Ar-filled glove box, where both O₂ and H₂O levels were maintained below 0.1 ppm. 1 M LiTFSI consisting of 1,3-dioxolane/1,2-Dimethoxyethane in a volume ratio of 1:1 with 2 wt.% lithium nitrate (1 M LiTFSI in DOL/DME with 2 wt.% LiNO_3_) was used as the electrolyte. To evaluate the ionic conductivity (σ) of the separator soaked in the electrolyte, SS||SS cells were tested using electrochemical impedance spectroscopy (EIS) within a frequency range of 10⁶ to 1 Hz on a CHI760E electrochemical workstation. The ionic conductivity was determined using the following equation:

$\sigma=d/RS$ (1)

Where *d* was the thickness of the separator, *R* and *S* corresponded to the bulk resistance and the effective area of the separators, respectively.

The transference number of lithium ions (*t_Li_^+^*) was determined using both AC impedance and DC potentiostatic polarization measurements with a Li||Li symmetric cell on an Autolab PGSTAT302N electrochemical workstation. During the DC polarization, a small voltage (10 mV) was applied for a sufficient duration to allow the current to reach a steady state. The interfacial resistances of the cells were measured both before and after polarization, and were estimated by analyzing the diameter of the semicircle in the high-to-medium frequency range of the impedance spectrum. The *t_Li_^+^* was calculated according to the following equation:

$t_{Li}^{+}=I_{s}\left( \Delta V-I_{0}R_{0} \right)/I_{0}\left( \Delta V-I_{s}R_{s} \right)$ (2)

where *ΔV* is the potential applied across the cell. *I_0_* and *I_s_* represent the initial state current and steady state current, respectively. *R_0_* and *R_s_* represent the initial state resistance and steady state resistance, respectively.

*Electrochemical Measurements*: To assess the oxidation limit of the separators in the redox environment of LMBs, LSV was conducted between 2.5 and 6 V at a scan rate of 1 mV s⁻¹ using Li||SS cells. Additionally, a galvanostatic test was performed on Li||Li symmetric cells, applying a current of 0.5 mA cm⁻² for 1 hour of charging followed by 1 hour of discharging. The LMBs were investigated using LiFePO_4_ as the cathode and metallic lithium as the anode. The cathode materials were fabricated by blending LiFePO_4_ powder, PVDF, and Super P in a weight ratio of 8:1:1, with N-methyl pyrrolidone (NMP) serving as the solvent. The slurry was then coated onto an aluminum foil collector and dried under a vacuum at 100°C for 12 hours. The charge and discharge tests of LiFePO_4_||Li cells were performed in the voltage range of 2.5 to 4.0 V at various current densities using a channel battery analyzer (NEWARE CT4008T). The cathode loading is 1.6 mg/cm², the electrolyte volume is 40 μL, and the lithium foil thickness is 450 μm.

*Calculations Methods*: The weak interaction energies between PDA and PDA(Cu) with DOL/DME solvent were calculated by using the Gaussian 16 package^[1]^. The model structures of PDA(Cu) and PDA with explicit solvent molecules around them, including two DOL and two DME molecules. Various structures for PDA(Cu) and PDA are constructed according to the electrostatic potential distribution, aiming to find the structures with the lowest energy. Then, the obtained structures with the lowest energy were optimized at PBE1PBE/def2tzvp theoretical level with a D3 version of Grimme’s dispersion with Becke-Johnson damping, where Lanl2DZ pseudopotential and basis set were adopted for the Cu^2+^. The subsequent energy calculation for the complex systems and separated solvent and solute were performed at PBE1PBE/def2tzvp theoretical level with a D3 version of Grimme’s dispersion with Becke-Johnson damping. The SDD pseudopotential and basis set were used for Cu^2+^ instead. SMD solvation model with appointed tetrahydrofuran solvent was utilized for all density functional theory (DFT) calculations in this work. The Multiwfn software was used to perform wavefunction analysis^[2]^, including interaction region indicator (IRI), independent gradient model (IGM), electron localization function (ELF), Mayer bond order and fuzzy bond order, Localized molecular orbital (LMO), and orbital composition analysis^[3, 4]^.

.

**Supporting Figures**


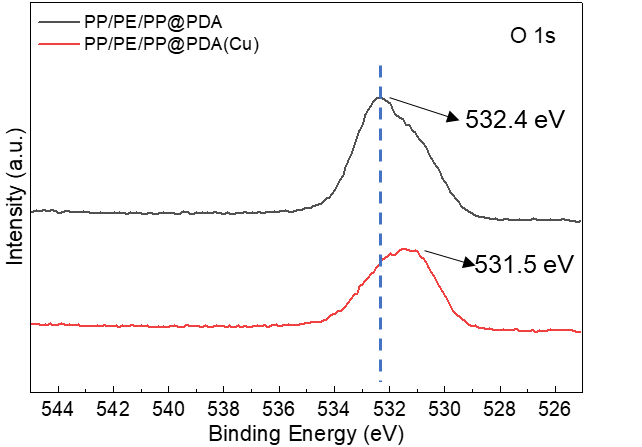


Figure S1. The O 1s XPS peaks of PP/PE/PP@PDA and PP/PE/PP@PDA(Cu).

Figure S2. EPR spectra of PP/PE/PP@PDA and PP/PE/PP@PDA(Cu).


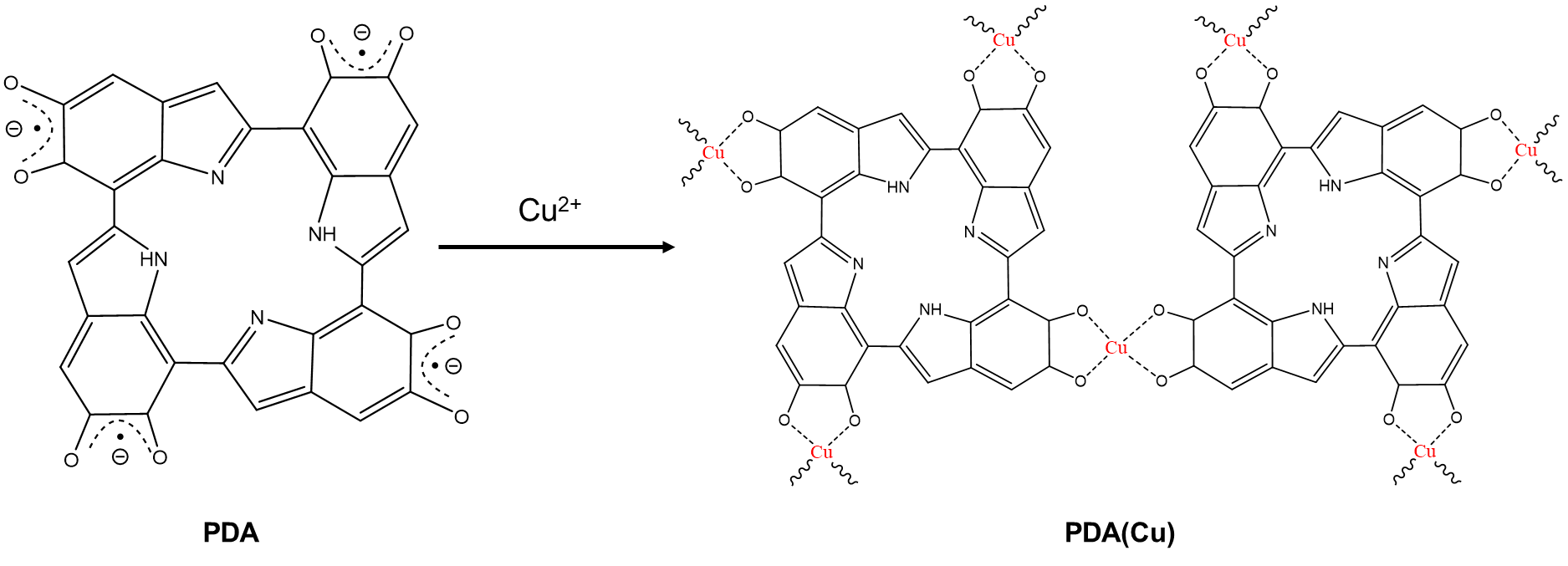


Figure S3. The chemical structure of PDA(Cu).


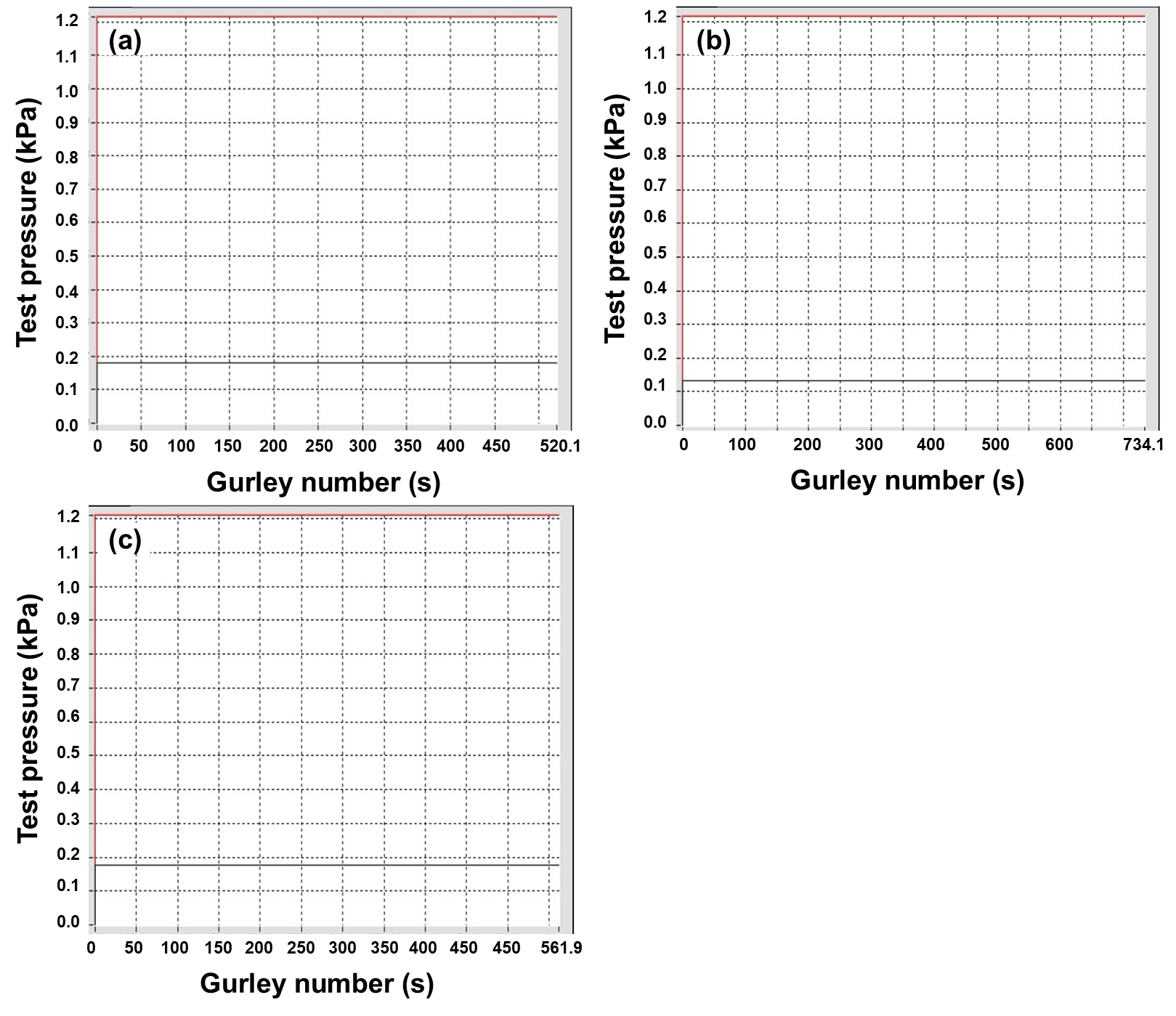


Figure S4. The air permeances of separators by the Gurley method: (a) PP/PE/PP; (b) PP/PE/PP@PDA; (c) PP/PE/PP@PDA(Cu).


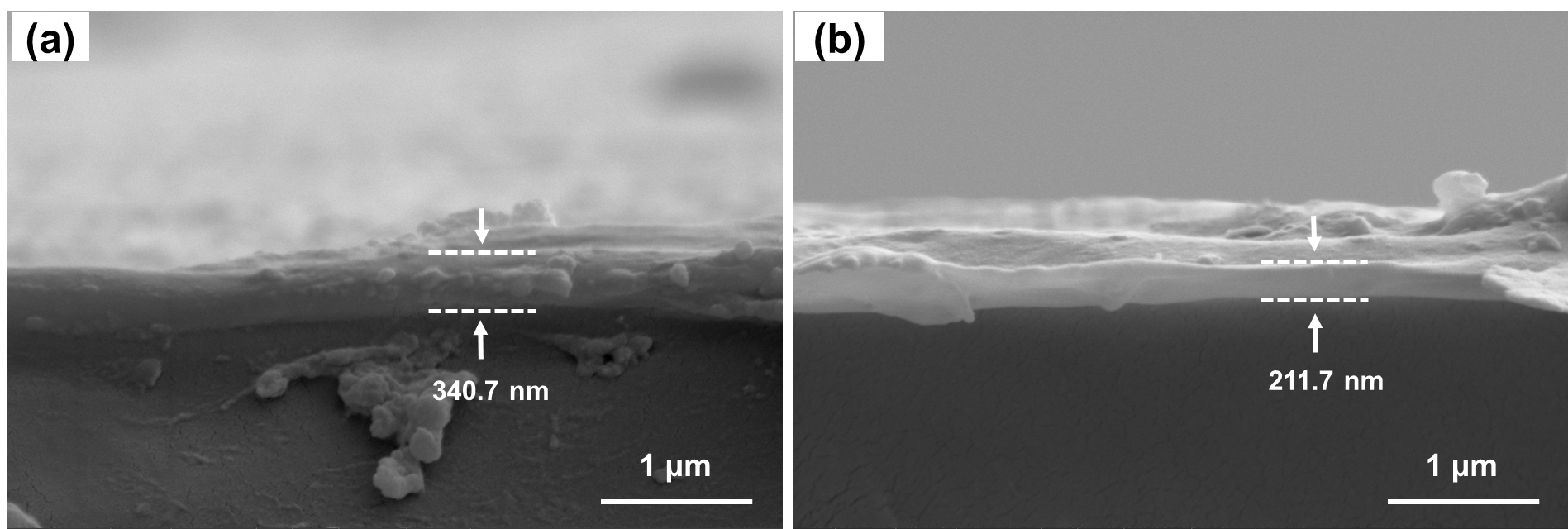


Figure S5. SEM photographs of the modified separators on cross-section: (a) PP/PE/PP@PDA; (b) PP/PE/PP@PDA(Cu). The values in the figure represent the thickness of the coatings.


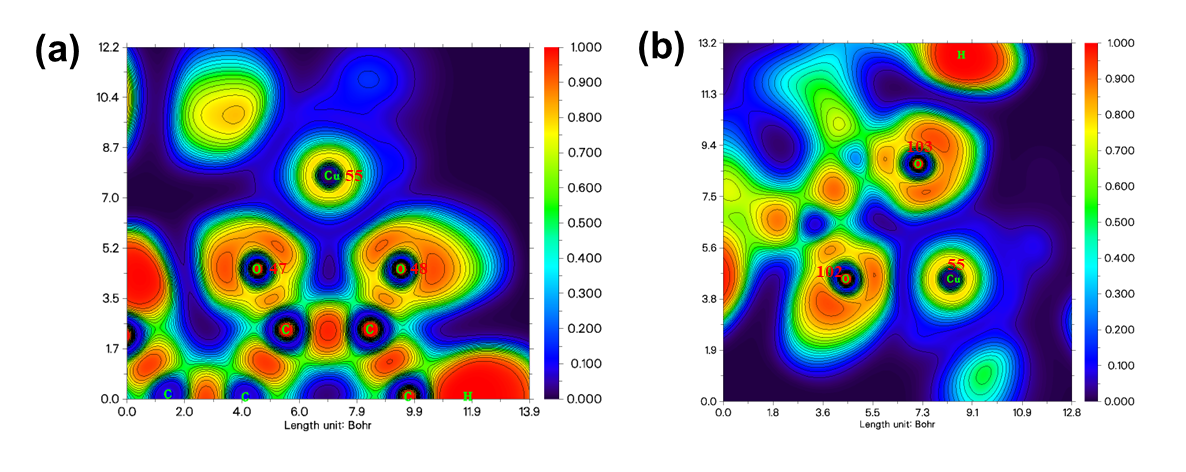


Figure S6. Electron localization function (ELF) of PDA(Cu) focusing on the Cu-center structure.

Table S1. Mayer bond order and fuzzy bond order of the Cu-center structure in PDA(Cu)

| Atom 1 | Atom 2 | Mayer bond order | fuzzy bond order |
| --- | --- | --- | --- |
| 47(O) | 55(Cu) | 0.31 | 0.70 |
| 48(O) | 55(Cu) | 0.30 | 0.63 |
| 102(O) | 55(Cu) | 0.44 | 0.88 |
| 103(O) | 55(Cu) | 0.21 | 0.48 |
| 145(O) | 55(Cu) | 0.29 | 0.70 |


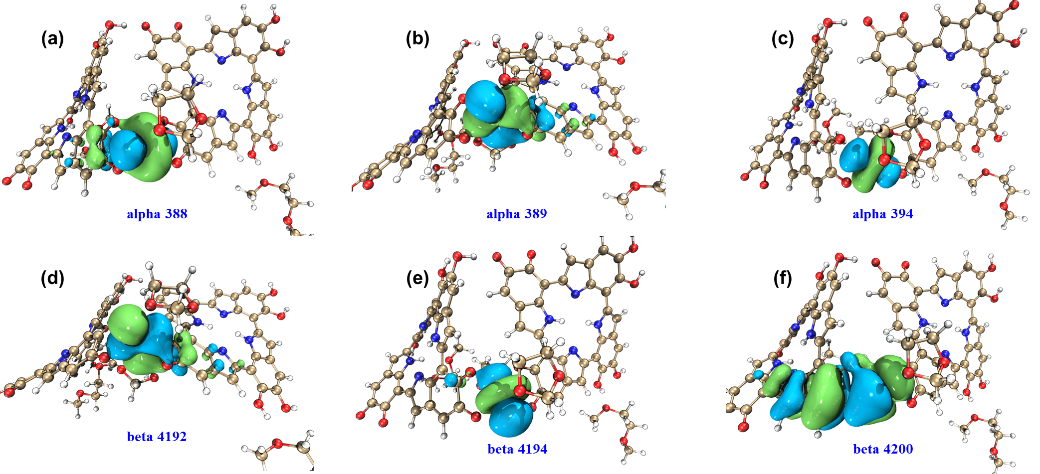


Figure S7. Localized molecular orbital (LMO) of the Cu-center structure in PDA(Cu): (a-c) alpha orbitals and (d-f) beta orbitals.

Figure S8. Nyquist plots of symmetrical SS||SS cells using the electrolyte with PDA depolymerization products (e-PDADP).


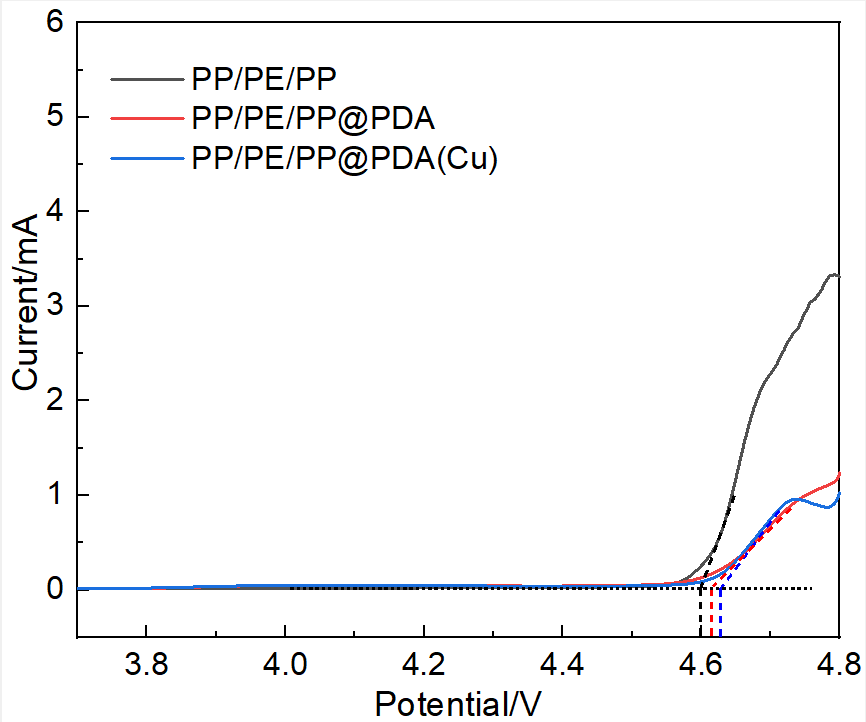


Figure S9. Linear sweep voltammetry (LSV) curves of SS||Li cells assembled with various separators.


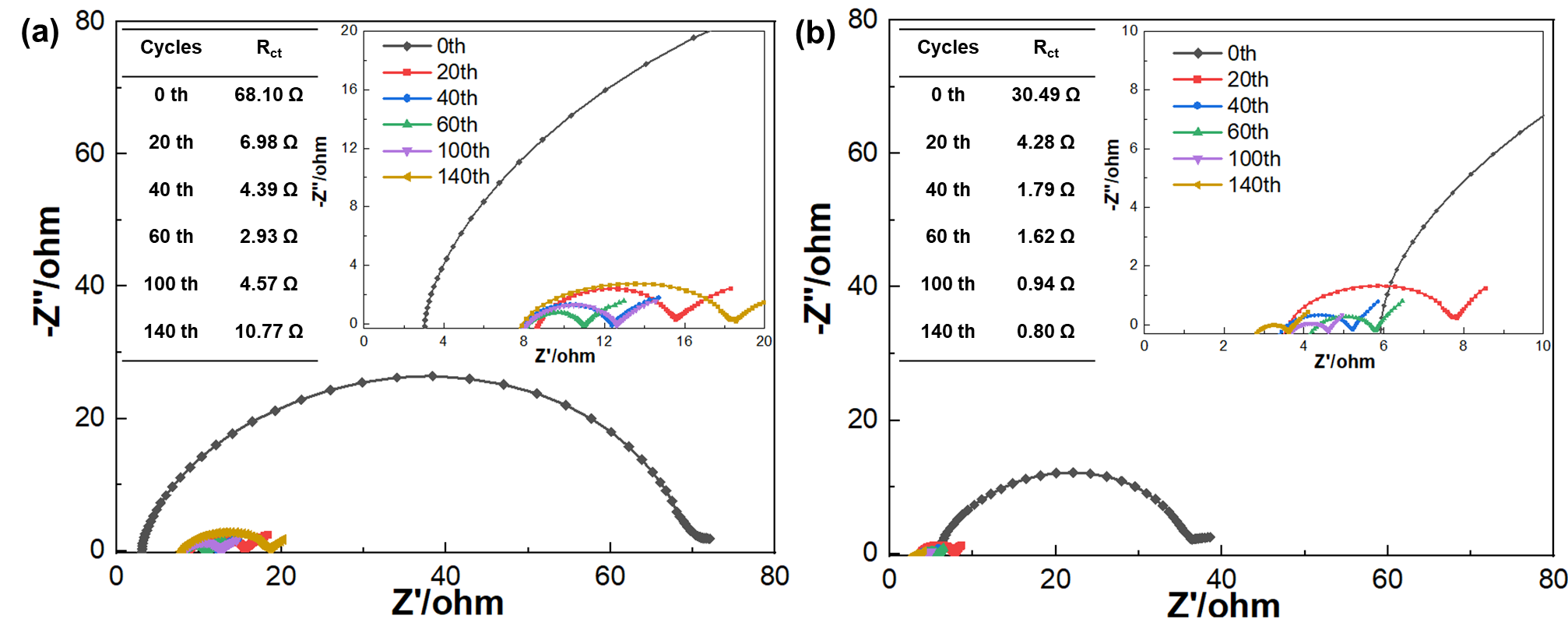


Figure S10. EIS spectra of Li||Li cells assembled with (a) PP/PE/PP; (b) PP/PE/PP@PDA(Cu) before cycling and after 20, 40, 60, 100, and 140 cycles.


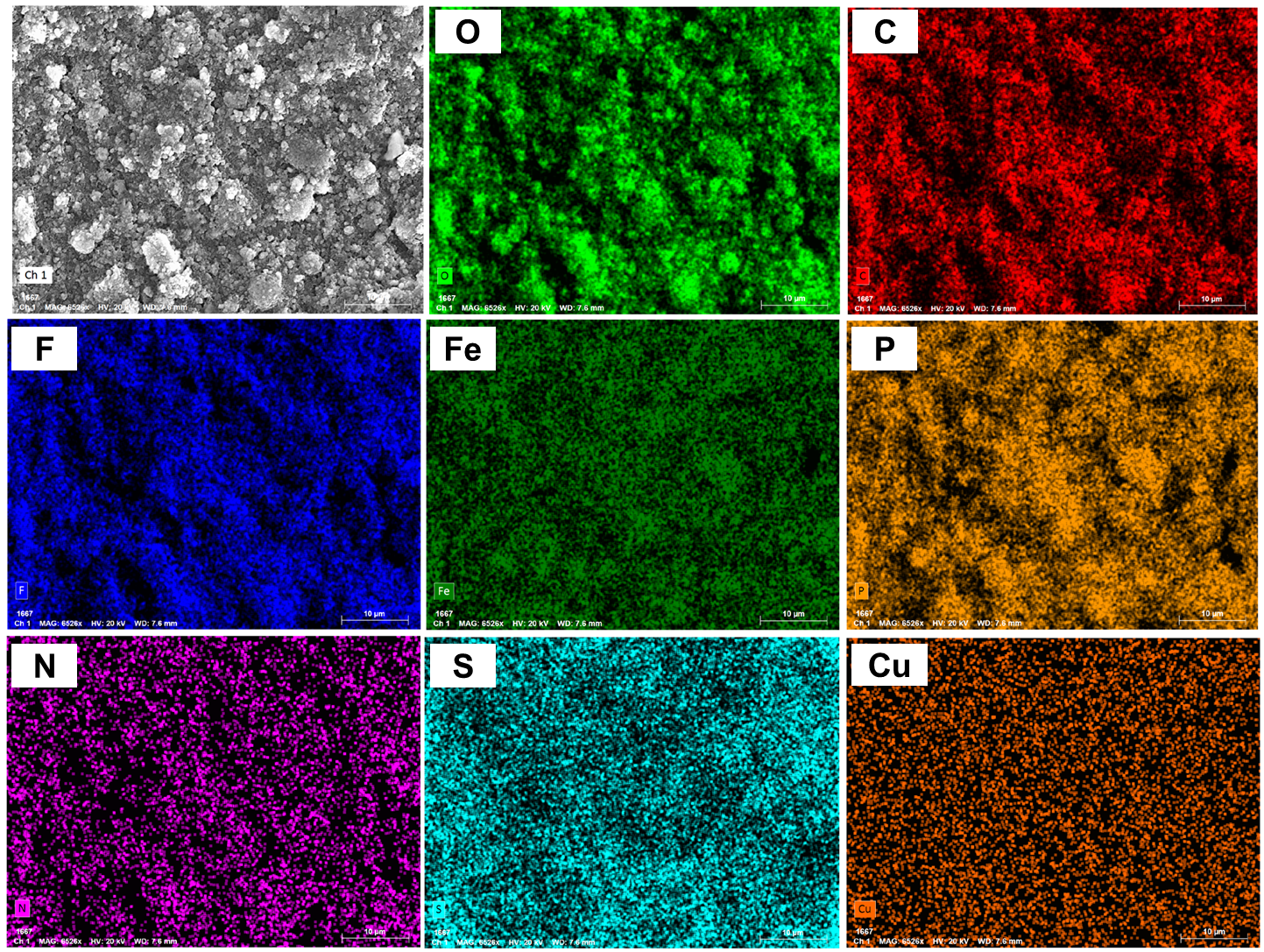


Figure S11. EDS results of the LiFePO_4_ cathode with PP/PE/PP@PDA(Cu) after 6000 cycles.


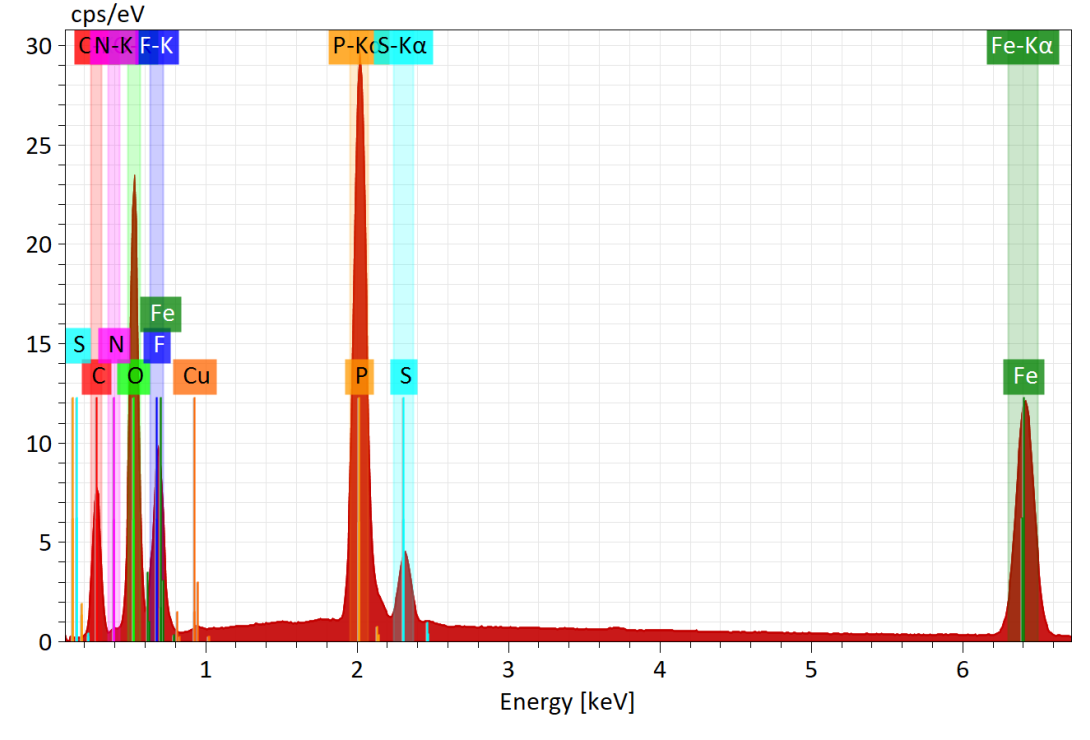


Figure S12. EDS spectra of the LiFePO_4_ cathode with PP/PE/PP@PDA(Cu) after 6000 cycles.

Table S2. Atomic contents of the LiFePO_4_ cathode with PP/PE/PP@PDA(Cu) after 6000 cycles

| Element | Atomic (%) |
| --- | --- |
| O | 37.65 |
| C | 35.68 |
| F | 9.41 |
| Fe | 6.64 |
| P | 6.47 |
| N | 3.14 |
| S | 0.92 |
| Cu | 0.09 |

**References**

[1] M. J. Frisch, G. W. Trucks, H. B. Schlegel, G. E. Scuseria, M. A. Robb, J. R. Cheeseman, G. Scalmani, V. Barone, G. A. Petersson, H. Nakatsuji, X. Li, M. Caricato, A. V. Marenich, J. Bloino, B. G. Janesko, R. Gomperts, B. Mennucci, H. P. Hratchian, J. V. Ortiz, A. F. Izmaylov, J. L. Sonnenberg, Williams, F. Ding, F. Lipparini, F. Egidi, J. Goings, B. Peng, A. Petrone, T. Henderson, D. Ranasinghe, V. G. Zakrzewski, J. Gao, N. Rega, G. Zheng, W. Liang, M. Hada, M. Ehara, K. Toyota, R. Fukuda, J. Hasegawa, M. Ishida, T. Nakajima, Y. Honda, O. Kitao, H. Nakai, T. Vreven, K. Throssell, J. A. Montgomery Jr., J. E. Peralta, F. Ogliaro, M. J. Bearpark, J. J. Heyd, E. N. Brothers, K. N. Kudin, V. N. Staroverov, T. A. Keith, R. Kobayashi, J. Normand, K. Raghavachari, A. P. Rendell, J. C. Burant, S. S. Iyengar, J. Tomasi, M. Cossi, J. M. Millam, M. Klene, C. Adamo, R. Cammi, J. W. Ochterski, R. L. Martin, K. Morokuma, O. Farkas, J. B. Foresman, D. J. Fox, Wallingford, CT, 2016.

[2] T. Lu, F. Chen, *J. Comput. Chem.* **2012**, *33*, 580.

[3] C. Lefebvre, G. Rubez, H. Khartabil, J.-C. Boisson, J. Contreras-García, E. Hénon, *Phys. Chem. Chem. Phys.* **2017**, *19*, 17928.

[4] T. Lu, Q. Chen, *J. Comput. Chem.* **2022**, *43*, 539.
